# The Impact of Chronic Discogenic Low Back Pain: Costs and Patients' Burden

**DOI:** 10.1155/2018/4696180

**Published:** 2018-10-01

**Authors:** José W. Geurts, Paul C. Willems, Jan-Willem Kallewaard, Maarten van Kleef, Carmen Dirksen

**Affiliations:** ^1^Department of Anesthesiology and Pain Medicine, Maastricht University Medical Centre, Maastricht, Netherlands; ^2^Department of Anesthesiology and Pain Management, Rijnstate Hospital, Arnhem, Netherlands; ^3^Department of Orthopedic Surgery, Maastricht University Medical Centre, Maastricht, Netherlands; ^4^Department of Clinical Epidemiology and Medical Technology, Maastricht University Medical Centre, CAPHRI—Care and Public Health Research Institute, Maastricht, Netherlands

## Abstract

**Introduction:**

Chronic discogenic low back pain (CDP) is frequently diagnosed in patients referred to specialized pain clinics for their back pain. The aim of this study is to assess the impact of CDP both on the individual patient and on society.

**Materials and Methods:**

Using the baseline records of 80 patients in a randomized trial assessing the effectiveness of a new intervention for CDP, healthcare and societal costs related to back pain are calculated. Furthermore, the impact of the condition on perceived pain, disability, health-related quality of life, Quality of life Adjusted Life Years (QALY), and QALY loss is assessed.

**Results:**

Using the friction costs approach, we found that the annual costs for society are €7,911.95 per CDP patient, 51% healthcare and 49% societal costs. When using the human capital approach, total costs were €18,940.58, 22% healthcare and 78% societal costs. Healthcare costs were mainly related to pain treatment. Mean pain severity was 6.5 (0–10), and 46% suffered from severe pain (≥7/10). Mean physical limitations rate was 43.7; 13.5% of the patients were very limited to disabled. QALY loss compared to a healthy population was 64%.

**Discussion:**

This study shows that in patients with CDP referred to a pain clinic, costs for society are high and the most used healthcare resources are pain therapies. Patients suffer severe pain, are physically limited, and experience a serious loss in quality of life.

## 1. Introduction

Low back pain is one of the most common disabling conditions worldwide [1]. Approximately 70 to 85% of the western population will develop low back pain at least once during their lifetime [2]. Of the people that consult their general practitioner for low back pain, one year later about 60% still report pain [3]. The prevalence of chronic low back pain (CLBP) is globally calculated to be 9442.5 per 100,000 (9%) [4]. CLBP is a common, long-lasting, and disabling condition with high costs for society [5–7]. In 2007, the costs for CLBP represented 0.6% of the gross national product in the Netherlands [7]. Direct healthcare costs are, for instance, caused by patients searching for pain treatment [2, 5, 8, 9]. Indirect (societal) costs represent secondary consequences of CLBP, the losses resulting from morbidity or disability, mainly caused by work absenteeism and informal caregiving [10, 11]. Although indirect costs are known to be the highest cost factor for CLBP, direct healthcare costs, like medical specialist care and hospital costs, for low back pain are high as well [5, 8, 11].

CLBP may emerge from several different etiologies but about 40 to 50% of CLBP, treated in specialized pain or orthopedic clinics, is alleged to be of discogenic origin [12–14]. Disk degeneration involves structural disruption and cell-mediated changes in composition of the disk, particularly annular fissures reaching the outer annulus [15]. Provocation discography is, up to now, the only test that with some reliability can distinguish pain of discogenic origin from other sources of CLBP [15, 16]. Patients with chronic discogenic low back pain (CDP) in clinical practice differ from other CLBP patients in that the chronic pain is located more axial and the pain is severe [17]. There is evidence that CDP more often starts at a younger age than other types of chronic pain. A study reports that if the CLBP starts at a young age, the more likely the pain is discogenic in origin [14, 18]. Although most CDP patients are amenable for pain intervention therapies, up till now there is no evidence for a longer term beneficial treatment [19].

Little is known about the specific impact of CDP on patients and its burden for society. About the impact of CLBP in general, there is more information [12, 13, 20] and some is known about the impact of radicular syndromes [21].

Therefore, the first aim of this study is to provide healthcare and societal cost information about CDP patients who have been referred to pain specialized care. The second aim is to assess the impact of CDP on patients' pain, disability, health-related quality of life, and QALY loss.

## 2. Methods

### 2.1. Design

This study is a CDP evaluation with both a cost of illness analysis and assessment of outcome [22].

There are two methods available to estimate costs of disease. Top-down healthcare cost data can be obtained from central data collecting sources like medical insurance agencies [8, 23]. The other method is bottom-up data collection that uses individual patient-level information collected by surveys and diaries from a single study or multiple smaller studies. Our approach is the bottom-up method using data obtained in a multicenter study. This study was performed from 2013 to 2016 in 4 pain centres throughout the Netherlands. Patients with CLBP were predominantly referred from primary care, and sometimes from neurologic or orthopedic settings. Baseline data were collected during the IMBI RCT [24] that was conducted to assess the efficacy and cost effectiveness of a minimal interventional procedure for CDP. The diagnosis of CDP cannot be made with a reasonable amount of certainty with conventional clinical tests [13]. Therefore, data from this study were used to obtain information from CDP patients for whom a provocation discography had been performed to confirm the suspected diagnosis of CDP [17].

### 2.2. Participants

Eighty consecutive patients with CDP, as diagnosed by clinical history, physical examination, magnetic resonance imaging, and provocation discography, were eligible for the IMBI study and were included in this evaluation. Inclusion and exclusion criteria were described in detail in the study protocol of the RCT [24]. Patients had to be above 18, have a BMI of ≤ 35, and had to rate at least a 5 for their low back pain on an 11 box (0–10) numeric rating scale after at least 6 months of pain treatment. Facet pain was excluded by negative facet blocks. The diagnosis of discogenic pain was confirmed by a positive provocative discography along with morphologic signs of disc degeneration, that is, annular tear grade II to IV according to the modified Dallas Classification [24, 25].

### 2.3. Assessment of Impact

The baseline data used for this study included sociodemographic characteristics, pain severity and pain medication use, disability, and health-related quality of life [26]. Furthermore, cost information over the last 3 months, e.g., healthcare utilisation, medication, and lost working hours, was collected from each patient entering the study. Health-related quality of life at baseline and 3-month cost information were projected over a full year for calculation of QALY (loss) and annual societal costs.

### 2.4. Pain

Patients rated their low back pain in a pain diary, 3 times a day during 4 days, using 11 box (0–10) numeric rating scales (PNRS); zero represents no pain and 10 excruciating pain [26].

### 2.5. Disability

Physical functioning was measured with the Oswestry Disability Index (ODI) [27]. The value of the ODI scale represents the physical limitations rate (0–100%):

- 0–20% minimally limited: patient can get along with most daily activities. There is normally no treatment indicated except advice about sitting, lifting, and exercises.
- 21–40% moderately limited: patient experiences pain when lifting, sitting, and standing. Travel and social life is sometimes difficult, and absenteeism can occur. Normally, there are no limitations in daily activities, sexual activity, or sleeping.
- 41–60% clearly limited: pain is problem in activities. Treatment is indicated.
- 61–80% very limited to disabled: back pain affects all aspects of life of the patient. Treatment is very desirable.
- 81–100% disabled. These patients are often bedridden.

### 2.6. Health-Related Quality of Life

Health-related quality of life was assessed with the Rand-36 and the EuroQol (EQ-5D-3L) [28–30]. The Rand-36 measures eight domains of quality of life: physical functioning, social functioning, role limitations (physical problem), role limitations (emotional problems), mental health, pain, general health perception, and health change. Furthermore, two summary scores were calculated from the Rand-36: the physical component summary (PCS) and the mental health component summary (MCS). A higher score relates to a better health status. The EQ-5D-3L was used to measure health state, it contains 5 domains: mobility, self-care, usual activities, pain/discomfort, and anxiety/depression, and a general health score measured with a visual analogue scale 0–100 (VAS) [31, 32]. Each domain has three levels: no limitations, some limitations, and severe limitations. This results in 243 possible sets of health states. A selection of these health states has been valued by the general public in the U.K. using the time-trade-off method. This resulted in an algorithm which allows calculation of a utility value (i.e., quality of life score) for each possible health state [31]. The utility values range from −0.549 to 1, where 1 is perfect health and ≤ 0 is a health state equal to death or a health state considered worse than death.

### 2.7. Quality of Life Adjusted Life Years (QALY)

The QALY is a composite measure which multiplies life years with the quality of these life years. One QALY represents one life year in perfect health. QALYs lived in one year were calculated based on the EQ-5D-3L at baseline, using the UK algorithm [31]. It was assumed that the utility score at baseline represented the utility score for a full year. Furthermore, because CDP is not a fatal disease, it was assumed that the QALY loss was fully attributed to the loss in health-related quality of life.

### 2.8. Costs

Health care and societal costs were measured with cost questionnaires with a recall period of 3 months, which were filled out by patients online [33].

The patients were asked to record the resource use specifically related to their treatment of back pain, such as visits to primary care, medical specialists, physical therapists, and complementary and alternative medicine. Furthermore, the questionnaire queried specifically about back pain-related medication costs, extra requirements such as adaptations to home or equipment for their mobility, and professional caregiving costs. Moreover, patients were asked to report societal costs that included lost productivity and informal caregiving costs. Work absenteeism was measured with the productivity and disease questionnaire (PRODISQ) [34].

### 2.9. Statistical Methods

With the exception of the pain diary and the EQ-5D-3L, which were presented to the patient in a booklet, all data were collected by web-based questionnaires software, SelectSurvey (NETv4.075.011© Copyright 2008 ClassApps.com) and MACRO (version 4.1.2.3750© 1999–2012 InferMed Limited, London, UK) [24]. Before the intervention, the booklets were inspected for missing data, and completion of the online questionnaires was checked. After these preparations, we assumed that patients filled in the relevant questions correctly, and no correction was made for missing baseline data of the 80 patients used for this study. Costs were calculated by multiplying resource use by the cost price using Dutch guideline prices for the resources, reference year 2014 [35]. If a guideline price was not readily available, an assumption was made on the basis of an existing guideline price. We used mean hourly labor costs for employees across all sectors of €37.50 [35]. Informal care and unpaid productivity loss was valued at €14/hour [35].

All costs-related information from the baseline questionnaires in which patients were asked about information covering a period of 3 months before entering the study were multiplied by 4 to calculate annual costs per patient. Study-related costs, like costs made for informed consent consults, were not included in the cost analysis. The cost of work absence (productivity losses) was calculated using the friction cost method, which assumes that each worker is replaceable within 85 workdays or 12 weeks [6, 11, 35, 36]. Additionally, the human capital approach was used, as this method is applied in many cost studies as well [6, 37]. Both approaches assume that the individual's level of earnings reflects their productivity. However, in the human capital approach, lost productivity due to long-term absenteeism is valued, even until the age of retirement [10].

In this study, we estimated the costs of absenteeism due to CDP for each patient by multiplying the total number of sick days by the mean number of daily working hours and the costs per hour [35]. Using the human capital approach, disability wages were calculated to be 75% of the average working costs per hour with a maximum of €52.766 a year according to the Employee Insurance Agency (UWV) in the Netherlands [38].

Results are presented as means, range, and standard deviation. Costs results are presented as bootstrapped means and 95% confidence intervals of means (CI). Statistical analyses were performed using SPSS version 24 (SPSS Inc., Chicago IL, USA).

## 3. Results

**Figure 1:**
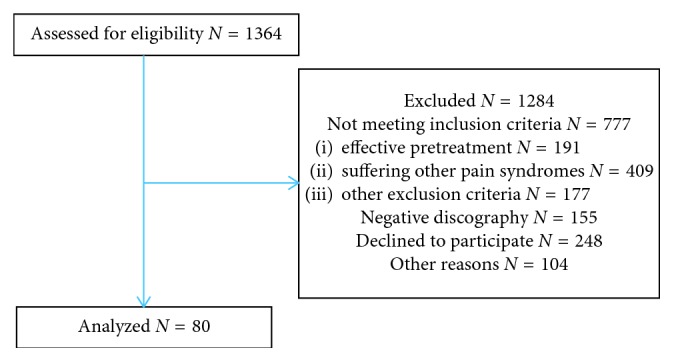
Flow diagram.

Eighty patients included in the IMBI study [24] were assessed for baseline information.  Figure 1 shows the flow diagram of the IMBI study. More than 1300 patients were assessed for eligibility in the participating centres. Fifty-seven percent did not meet the inclusion criteria. More than 50% of discographies were negative.  Table 1 shows the patient characteristics. The study group comprised 57 (71%) female and 23 (29%) male patients. Mean age was 42 (21 to 65). Mean BMI was 25.3 (18 to 35), length 174 (154 to 196) cm, and weight 77.3 (42 to 118) kg.

### 3.1. Pain

Table 1 shows that the patients scored the severity of their low back pain on mean at 6.5 (PNRS: 0 to 10). Maximum mean pain was 8.8 (SD: 1.0). Low back pain duration was mean 10.6 (1 to 40) years.

### 3.2. Disability

The mean physical limitations rate was 43.7 (SD: 14.9) as measured by the ODI [27]. All patients showed some physical limitations of their CDP. Thirty-seven patients (46%) had clear physical limitations, 30 (38%) showed moderate limitations, 9 (11%) patients were very limited to disabled, and 2 (2.5%) patients were disabled by the CDP. Two patients (2.5%) had minimal physical limitations.

### 3.3. Health-Related Quality of Life

**Figure 2:**
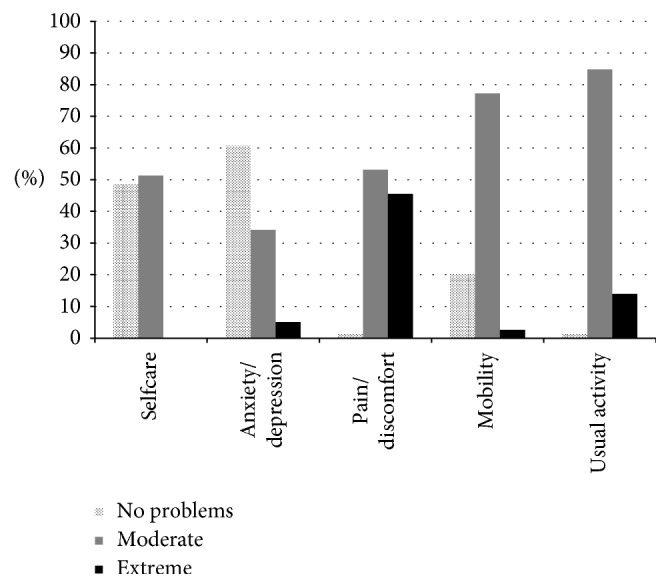
Baseline EQ-5D health profile (percent patients' response categories per domain).

Health-related quality of life as measured with the Rand-36 and EQ-5D-3L is shown in Table 2 and Figure 2. Using the EQ_5D_3L, when perfect health is scored at 100 and death is scored at zero, this CDP population scored on mean 52.5 (SD: 17.7). Figure 2 compares the outcome of the 5 health subscores of the EQ-5D-3L. This figure shows that most CDP patients score on average low on the domains pain, mobility, and usual activity. The Rand-36 shows the same results. The lowest scores were shown on the item “role limitations due to physical functioning” mean 15.3 (SD: 26.3) and bodily pain 32.9 (SD: 17.2).

### 3.4. Quality-Adjusted Life Years (QALY) and QALY Loss

Table 1 shows that the mean utility for the health state of CDP patients was 0.36 (SD: 0.34), resulting in a mean QALY of 0.36. Compared to a year in full health, this can be translated as a QALY loss of 64% [39].

### 3.5. Costs

Table 3 shows the mean CDP-related healthcare costs. Mean annual costs were calculated to be €4,015.38. Patients' visits for their back pain to the daycare clinic generated the most costs, mean €1,955.00 (CI: 1,565–2,353) per patient followed by back pain-related consults physical therapy, i.e., €461.10 (CI: 245–676).

Sixty-seven patients (84%) regularly used analgesics for their low back pain: 33% of patients regularly used paracetamol, 31% used nonsteroidal anti-inflammatory drugs, 14% used weak opioids analgesics, and 11% of patients used strong opioids. The mean yearly cost for medication was calculated at €144.56 (CI: 89–200). Approximately 80% of healthcare cost are targeted at pain therapies and related therapies like physical therapy (12%), psychosocial therapy (6%), and 4% for complementary and alternative medicine (CAM). Daycare clinic and pain clinic both made up for 53.5% of the healthcare costs.

The total mean societal costs were composed out of informal caregiving and costs related to work absence (see Table 4). Thirty-seven (46%) patients reported costs for informal caregiving, and 20 patients (25%) noted work absence. The mean absence from work (friction) costs per patient was €3,778.32 versus €14,806.95 using the human capital approach.

Using the friction cost approach, the mean annual total costs per patient with CDP were €7,911.95: €4,015.38 (51%) healthcare cost and €3,896.57 (49%) societal costs.

When the human capital cost approach is used, the total mean annual cost per CDP patient was calculated to be €18,940.58: €4,015.38 (22%) healthcare cost and €14,925.20 (78%) societal costs.

## 4. Discussion

The main objective of this study was to assess the costs of CDP for patients and society. Furthermore, we assessed the impact of CDP on patients' pain, disability, health-related quality of life, QALYs, and QALY loss.

Almost half (46%) of CDP patients reported severe pain (>7 of PNRS 0 to 10), 54% suffered moderate pain. A European prevalence study in chronic pain suffering patients showed that 34% had severe pain and 66% had moderate pain [40]. In agreement with clinical practice, this study shows that CDP patients suffer more severe pain in relation to the general chronic pain suffering patients [40].

In this study, we found that the annual cost per patient for the society is €7,911.95: €4,015.38 (51%) healthcare cost and €3,896.57 (49%) societal costs, using the friction costs approach. Using the human capital approach, the mean cost is €18,940.58: €4,015.38 (22%) for the healthcare cost and €14,925.20 (78%) societal costs per patient.

Costs are on average 56% lower in studies using the friction cost approach than in studies using the human capital approach, because it takes into account that employees can be replaced after a certain time period, i.e., for this study, 85 workdays or 12 weeks [11, 36]. In contrast with the human capital method, long-term absenteeism and disability do not induce additional costs when applying the friction cost method.

The cost for disability wages (€6,424.03) was only considered in the human capital approach [41]. Including work disability in the estimation of productivity costs was done with the assumption that the disability was caused by CDP. Although one of the basic principles of the human capital method is the assumption that there is full use of labor (i.e., no unemployment), the policy in the Netherlands is to provide for 75% of the last earned wages, and therefore, for the disability cost calculations, 75% of the mean hourly labor costs was used instead of 100%.

The inclusion and exclusion criteria for the RCT, from which this study retrieved information, could have an effect on the outcome considering that the patients with more than 35 BMI, or degeneration of multiple discs (>2) were excluded. Furthermore, patients with very severe disc degeneration (Grade V Modified Dallas Scale) were excluded in the RCT, and this study used data from [24, 25]. It is therefore likely that the burden and costs are even higher for the total CDP suffering population. This study shows that almost all CDP patients were physically disabled. This was not only shown with the ODI, but also the domain measuring role limitations physical functioning of the Rand-36 showed a very low score, 15.3 (SD: 26.3).

In comparison, a study in rheumatoid arthritis [42] patients showed a score of 48 for this domain, and in migraine suffering patients showed 60 in role physical functioning [29]. Furthermore, the domain physical functioning in our study was 48.3 (SD: 18.3), and a Dutch patient group suffering rheumatoid arthritis scored 62.3 (SE: 2.0) for physical functioning [42].

The HRQoL was not only low in the scores for physical functioning but also general health and bodily pain scores were low. The mean utility value (i.e., quality of life score) based on the EQ-5D was extremely low (0.36) in the CDP patients. General population score of people in Europe around 45 years of age is approximately 0.85; for instance, a chronic low back pain population in Finland scored mean 0.74 [43]. Similar low quality of life utility scores were reported in a population with major depressive disorders, 0.33 versus 0.36 in our population [44].

The lowest scores of the Rand-36 were shown on the item “role limitations due to physical functioning” mean 15.3 (SD: 26.3) and bodily pain 32.9 (SD: 17.2). In comparison, the Dutch general population scored, respectively, 79.5 (SD: 35.4) and 80.5 (SD: 24.4) [29]. Overall, the burden of disease resulted in a QALY loss of 64% compared to a person in full health.

Our study showed that the patients with CDP in our study suffered moderate to severe pain for on average 10 years (Table 1), are physically disabled, and have a low quality of life due to the bodily pain and functional limitations. This study also shows that unemployment is high in the CDP population: 16% in women and 17% in men (Table 1). On average, in the Netherlands 5% of the working population is unemployed according to the CBS Statistics, the Netherlands (CBS). Eleven (14%) patients in our study population received disability wages (DIA), 22% of the males and 9% of the females. In the Netherlands, on average 10% of the population receives DIA (CBS).

This study was performed in 4 pain centres across the Netherlands: 3 general hospitals and 1 university pain clinic. Only patients with a confirmed diagnosis of chronic discogenic low back pain were included in this study. Our study comprised 57/80 (71%) female patients. Although this seems a disproportional amount of the female gender, research shows that a larger part of the female CLBP suffering population has back pain with impairment: 70% in women versus 57% in men [2]. Our study records showed that apart from women having a longer treatment history (Table 1), mean 6.6 years in women and 3.5 in men, patient characteristics showed no obvious differences between the sexes.

The prevalence of CLBP is estimated to be 9% [4] in the general population, and approximately 50% of CLBP patients in a pain clinic are alleged to suffer discogenic pain [12–14]. However, this does not mean that the population prevalence of CDP is 4.5% (half of the CLBP population prevalence of 9%) because not all CLBP patients are referred to a pain clinic. It is likely that the average CLBP patient referred to a pain clinic is suffering more, and less episodic, severe pain. Therefore, we assume that the incidence of CDP within the CLBP of a pain clinic population is higher than in the general population. A recent study confirms this assumption by showing that only 13% of the CLBP in a nonpain clinic (an outpatient orthopedic clinic) could be diagnosed with discogenic pain [45]. Because the prevalence of CDP in the general population is uncertain, no efforts were made to extrapolate the costs to population level.

As far as we know, this is the first study that shows the economic burden for society and the impact of CDP on patients. A comparable study with bottom-up information conducted with patients from a pain clinic showed that the total costs of chronic pain suffering patients was US$24,043 using the human capital cost approach [46]. In this study, the hospital stay (nights) took the largest share of healthcare costs (44%). For CDP patients in our study, hospital nights due to CDP are rare, i.e., mean €59.50 (1%) per patient (Table 3). In contrast with the aforementioned study, more costs are made by our study population for therapies like physical therapy, psychotherapy, pain medication, and CAM therapies. Another study assessing cost effectiveness of steroid injections in radicular pain syndrome showed that the mean societal (friction) costs were €4,414 to €6,943 [21].

On average, CDP patients are in the middle of their productive life at the age of 40, have growing up children, have to pay off mortgage and plan ahead for the studies of their children. Patients suffering low back pain tend to return to work within 6 weeks to 3 months after the acute phase of the condition [38]. However, this does not always mean that the back pain suffering has stopped [3], and most CDP patients remain searching for an effective pain treatment. Our study shows that patients reported CLBP duration for a mean of 10.6 (1 to 40) years and pain treatment of on average six years. Despite all treatment received, before inclusion into the study assessing efficacy of a new treatment, the mean pain was still high (mean PNRS 6.5 (11box)) [24].

Although many healthcare resources are used (and much money is spent each year), the patients included in this trial at baseline still suffer pain and experience loss in quality of life. Innovations and further development of effective treatments are essential to better manage CDP and diminish associated patient burden and societal costs in the future.

## 5. Conclusion

Society spends €7,911.95 (friction costs approach) or €18,940.58 (human capital approach) each year per CDP patient. Societal costs are for a large part caused by work absence. In patients with CDP, healthcare resources are mostly used for pain therapies. Despite these efforts, baseline patients for this study suffer severe pain, are physically limited, and experience serious loss of quality of life.

## Figures and Tables

**Table 1:** Patient characteristics.

	*N* = 80	Male	Female
Gender, *N* (%)		23 (29%)	57 (71%)
	Mean (SD)	Mean (SD)	Mean (SD)
Age (years)	41.8 (9.9)	41.5 (8.7)	41.9 (10.5)
Low back pain (0–10)	6.5 (1.5)	6.7 (1.7)	6.4 (1.4)
Pain duration (years)	10.6 (8.0)	9.7 (5.6)	10.9 (8.8)
Treatment duration (years)	5.7 (5.7)	3.5 (2.5)	6.6 (6.3)
Education, *N* (%)			
Low	8 (10%)	2 (9%)	6 (11%)
Middle	45 (55%)	14 (61%)	31 (54%)
High	27 (35%)	7 (30%)	20 (35%)
Employment/work, *N* (%)			
Full time	31	12 (52%)	19 (33)
<30 hours a week	26	2 (9%)	24 (42%)
Unemployed	13	4 (17%)	9 (16%)
Disability payment (DIA)	10	5 (22%)	5 (9%)

Data presented as total group and divided by gender. DIA = Disablement Insurance Act; SD = standard deviation.

**Table 2:** Health-related quality of life of patients with discogenic low back pain.

	Mean	Std. deviation
*Rand (36)*		
Physical functioning	48.25	18.25
Social functioning	52.37	23.60
Role limitations physical	15.31	26.23
Role limitations emotional	59.16	43.40
Mental health	65.10	20.08
Bodily pain	32.88	17.22
Vitality	44.62	17.92
General health	35.62	20.96
*Physical sum score*	32.52	6.05
*Mental sum score*	44.67	10.96
*EQ-5D-3L*		
EQ-5D overall health (VAS)	52.51	17.74
UK utility value (EQ-5D)	0.36	0.34

VAS = visual analogue scale.

**Table 3:** Healthcare costs per CDP patient in euros divided by cost type.

Cost type	*N* visits^e^	*N* patients	Mean^ costs per resource user (CI)	Mean^ cost per patient^*∗*^ (CI)	Cum. %
Daycare clinic	340	53	2,950.94 (2574–3328)	1,955.00 (1565–2353)	48
Physical therapy	1112	27	1,475.52 (965–1986)	461.10 (245–676)	60
Travel costs	964	79	303.03 (232–374)	299.24 (229–370)	67
Psychosocial therapy	208	17	1,703.63 (615–2792)	234.25 (51–417)	73
Pain clinic	888	60	292.37 (227–358)	219.27 (163–275)	78.5
CAM therapies	224	10	1,330.80 (427–2234)	166.35 (30–303)	82.5
Medication	126	67	172.62 (108–237)	144.56 (89–200)	86
Extra requirements	36	9	1,128.89 (−36 to 2294)	127.00 (−6 to 260)	89
Rehabilitation	264	4	2,524.50 (−2273 to 7322)	126.23 (−53 to 305)	92
Home care	1056^h^	37	3.210 (1490–5010)	118.25 (−3 to 325)	95
Occupational physician	228	29	259.45 (173–345)	94.05 (53–135)	97
Primary care	164	18	300.67 (220–381)	67.65 (35–100)	98
Hospital nights	40	10	528.89 (407–651)	59.50 (20–99)	99
Policlinic other	92	4	523.25 (−852 to 1899)	26.16 (−19 to 72)	99.5
Intensive care	4	1	2,015	25.18 (−24 to 75)	100
Total healthcare costs		80	4,015.38 (3251–4779)	4,015.38 (3251–4779)	

^e^Visits/events/services/volumes; ^h^volume in hours; ^bootstrapped mean; ^*∗*^mean costs per patient = costs for all patients including cost per patients without visit/event/service (0€); CI = 95% confidence interval for mean: lower bound-upper bound.

**Table 4:** Societal costs per patient in euros divided by cost type (results of the friction cost and human capital cost approach).

Cost type friction costs approach	*N* ^e^	Mean^ costs per resource user (CI)	Mean^ cost per patient^*∗*^ (CI)	Cum. %
Informal caregiving	37	255.68 (143–368)	118.25 (60–177)	3
Absence work; friction costs	20	15,113 (11090–19135)	3,778.32 (2038–5518)	100
Societal cost per patient: Friction			3,896.57 (2157–5636)	
*Cost type human capital approach*				
Informal caregiving	37	255.68 (143–368)	118.25 (60–177)	0.8
Absence work; human capital	31	38,212 (28520–47903)	14,806,95 (9283–20330)	100
Disability wages	11	46,720 (39447–53994)	6,424.03 (2720–10128)	
Societal cost per patient: human capital			14,925.20 (9389–20460)	

^e^Number of patients per event/service; ^bootstrapped mean; ^*∗*^mean costs per patient = costs for all patients including cost per patients without event/service (0€); CI = 95% confidence interval for mean: lower bound-upper bound.

## Data Availability

The data used to support the findings of this study are available from the corresponding author upon request.
